# An Adaptive Multi-Channel Cooperative Data Transmission Scheduling in VANETs

**DOI:** 10.3390/s20195612

**Published:** 2020-10-01

**Authors:** Benhong Zhang, Baorui Yuan, Xiang Bi, Zhenchun Wei, Mingyue Zhang

**Affiliations:** 1 School of Computer Science and Information Engineering, Hefei University of Technology, Hefei 230009, China; zhangbh@hfut.edu.cn (B.Z.); ybrui@mail.hfut.edu.cn (B.Y.); bixiang@hfut.edu.cn (X.B.); 2019110994@mail.hfut.edu.cn (M.Z.); 2 Intelligent Interconnected Systems Laboratory of Anhui Province, Hefei University of Technology, Hefei 230009, China

**Keywords:** Internet of Vehicle, data transmission scheduling, multi-channel, ISing model

## Abstract

The Internet of Vehicle (IoV) technology is one of the most important technologies of modern intelligent transportation. The data transmission scheduling method is a research hotspot in the technology of IoV. It is a challenge to ensure the stability of data transmission due to fast network topology changes, high data transmission delays, and some other reasons. Aiming at the above problems, a multi-channel data transmission cooperative scheduling algorithm is proposed. First, construct a feasible interference map based on the data items sent and received by vehicles in the road scene. Second, assign channels to the nodes in the interference map based on the Signal-to-Interference-Noise-Ratio (SINR). Finally, the optimal multi-channel data transmission cooperative scheduling scheme is achieved through the ISing model. Simulation results show that compared with the traditional algorithm, the network service capacity is increased by about 31% and the service delay is reduced by about 20%. It ensures that emergency data is preferentially transmitted to the target vehicle and the maximum weighted service capacity of the network.

## 1. Introduction

Vehicular Ad-hoc Networks (VANETs) is the basis of modern intelligent transportation systems. It supports the wireless communication between Vehicle-to-Infrastructure (V2I) and Vehicle-to-Vehicle (V2V) [1]. Effective data transmission scheduling is the most basic guarantee to ensure personnel safety and smooth traffic. Due to the special nature of the Internet of Vehicles (IoV), there may be many challenges to ensure the successful transmission of data. The high mobility of the vehicle and the high data transmission delay lead to a shorter connection time and frequent communication confusion between road-side unit (RSU) and vehicles [2,3]. Data transmission scheduling is widely used to alleviate problems such as small RSU coverage and short vehicle connection time.

The vehicle transmission information in VANET mainly includes emergency information and status information, among which emergency messages include road accident warning and traffic signals [4,5]. Emergency messages such as traffic accidents are highly time sensitive and need to be transmitted to the required vehicles before the deadline, so higher requirements are placed on the stability of data transmission scheduling.

This paper studies that the cooperative data transmission scheduling problem in the hybrid V2I and V2V communication environment. In the RSU coverage area, some vehicles can obtain data items from RSU, that another part of vehicles can obtain data from their neighboring vehicles through V2V communication. Due to the limitations of RSU broadcast data items, the performance of data transmission can be enhanced by using the capacity of V2V communication. Vehicles sharing data using V2V communication can reduce the redundancy of using V2I communication to broadcast the same data item, thereby improving RSU bandwidth efficiency. In addition, through the reusability of space, the vehicle V2V communication can distribute multiple data items at the same time without interference. In particular, the main contributions of this paper are outlined as follows:

- This paper studies the data dissemination scheduling problem of V2I and V2V cooperative communication in a multi-channel environment maximize the weighted service capacity of data transmission. The data items are given a weight indicating the degree of urgency to ensure that the emergency data items are transmitted to the target vehicle before the deadline;
- We construct the interference graph based on the data dissemination cooperative scheduling problem and proposed the multi-channel cooperative data transmission scheduling (MDTCS) algorithm to solve the graph. The first part of the MDTCS algorithm is a greedy algorithm based on Signal-to-Interference-Noise-Ratio (SINR) to assign different channels to the vertices in the graph to solve part of the interference, which effectively reduces the waste of channel resources. The second part is a greedy selection algorithm based on the ISing model to solve the maximum weighted independent set in the scheduling graph, that is, the obtained independent set represents the optimal data transmission strategy;
- The simulation experiments prove that the performance of the proposed algorithm is better than some existing solutions.

The rest of this paper is organized as follows. Section 2 reviews the related work. Section 3 describes the system model and Section 4 describes the multi-channel data transmission cooperative scheduling process. Section 5 analyzes the performance of the proposed algorithm through simulation experiments. In Section 6 the whole paper is concluded.

## 2. Related Work

In recent years, research on the data transmission mechanism of the Internet of Vehicles mainly includes Media Access Control (MAC) protocols and data routing strategies [6]. Relevant research will be introduced separately below.

Guo et al. [7] proposed an adaptive collision-free MAC protocol based on a dynamic Time Divided Multiplexing Access (TDMA) mechanism. The RSU can dynamically maintain a list of vehicle allocation time slots in its coverage area. Vehicles transmit data according to the slot allocation scheme broadcast by RSU. Chen et al. [8] proposed a new adaptive coding strategy. This strategy uses effective data structures and pruning techniques to reduce the search space. Dang et al. [9] Proposed a MAC protocol that allows vehicles to broadcast secure data packets twice during the control channel interval and the service channel interval. Vehicles can send service packets within the control channel interval to improve service throughput. Gupta et al. [10] proposed a MAC protocol based on clustering cognition. When channel resources are insufficient, this protocol ensures the successful transmission of secure information at the cost of insecure information, thereby reducing delay and improving channel utilization. Nguyen et al. [11] proposed a hybrid TDMA/CSMA multichannel MAC protocol for VANETs that allows efficient broadcasting of messages and increases throughput on the control channel. Hwang et al. [12] proposed a lightweight data distribution protocol to solve the data distribution problem. By using the IEEE 802.11 management frame format for data transmission, there is no need to perform a link setting process in advance. Nguyen et al. [13] proposed a MAC protocol that can be applied to different traffic density. The Markov chain is used to study the multi-channel MAC protocol under saturated and unsaturated data traffic conditions, thereby reducing the service delay of data transmission. Karabulut et al. [14] proposed a novel orthogonal frequency division multiple access-based efficient cooperative MAC protocol for VANETs. Siddik et al. [15] propose a speed aware fairness-enabled MAC protocol that calculates the residence time of a vehicle in a service area by using mobility metrics such as position and speed to synthesize the transmission probability of each vehicle concerning its residence time.

The above introduction is about the research of data transmission in the MAC protocol. Data routing scheduling is also a research hotspot of the Internet of Vehicles. Liu et al. [16] proved the vehicle data transmission in the RSU coverage area as an NP-hard problem and then proposed a heuristic algorithm to solve the optimal transmission strategy, which effectively improved the network throughput. Azizian et al. [17] proposed a distributed clustered transmission scheduling scheme. This scheme provides contention-free transmission opportunities for sending vehicles by allocating time slots to different subsets, thereby improving network throughput. Zeng et al. [18] proposed a channel prediction data transmission scheduling algorithm. The least-square algorithm is used to predict the channel state to complete the data transmission scheduling. Wang et al. [19] proposed a dynamic clustering mechanism that considered the real-time position and speed of the vehicle. With RSU assistance, vehicles can dynamically join or leave clusters based on their real-time speed, which significantly improves the data transmission volume of vehicles in areas not covered by RSU. Dai et al. [20] proposed an adaptive scheduling (RCAS) algorithm based on RSU cooperation. The algorithm designed three mechanisms, including RSU centralized scheduling mechanism, vehicle temporary scheduling mechanism, and cluster management mechanism. Zhao et al. [21] proposed an optimization problem of minimizing the total number of time slots required for safety information sharing in VANETs. It narrows the feasible solution space through a clustering algorithm, which then minimizes the total number of time slots required for security information sharing in VANET. Benkerdagh et al. [22] performed data processing before the propagation process by building a fast and stable clustering scheme to improve message delivery time and obtain effective bandwidth consumption.

Data transmission scheduling on the Internet of Vehicles usually requires the assistance of RSU and the data transmission mode includes two communication modes: V2V and V2I. Busanelli et al. proposed a multi-hop probability broadcast protocol. In the highway scenario, the vehicle can only use the V2I communication mode to receive data items through RSU broadcast and by considering the physical characteristics of the network to ensure that the vehicle is not affected by traffic density when receiving data. The proposed algorithm does not consider data transmission scheduling in V2I and V2V mixed network environment. Liu et al. [23] proposed a single-channel Cooperative Data Dissemination (CDD) algorithm and used a greedy algorithm to solve the scheduling scheme. It is the first time that a centralized RSU is proposed to control the V2I and V2V coordinated communication data transmission, which proves that the coordinated data transmission scheduling problem is an NP-hard problem. Then the coordinated data transmission scheduling is transformed into the largest independent set in the interference graph. Finally, the RSU broadcasts to all vehicles to communicate correspondingly to improve network throughput. Peng et al. [24] proposed a Multi-Channel VANET-Data Transmission Scheduling (MCV-DTS) algorithm for multi-channel networking in a multi-channel environment. It constructs a conflict matrix based on the conflicted relationship between vehicle transmission requests. Then it is proved that the conflict matrix has positive semi-definiteness, that the semi-definite programming method is used for channel allocation to solve the optimal transmission scheduling scheme. It creates a conflict graph by asking a problem model and then solves the graph to obtain the optimal transmission scheme.

The above studies did not consider the urgency of the data and effective use of channel resources during vehicle data transmission. Based on the research of this problem, this article is dedicated to research on the priority transmission of emergency data and the optimal use of channel resources.

## 3. System Model

### 3.1. Network Model

**Figure 1:**
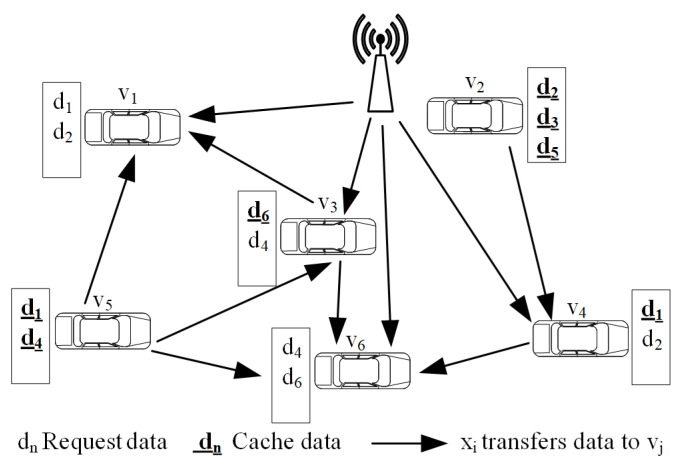
Coordinated scheduling graph of data transmission assisted by RSU.

The network model in this paper is shown in Figure 1. All vehicles are covered by RSU and there is one vehicle in the dispatch cycle. Each vehicle is equipped with a global positioning system and on-board unit, and the vehicle can obtain real-time position and speed. As the vehicle-mounted unit is half-duplex communication, it can only be in the sending or receiving state in the same cycle. Each vehicle contains a cached data item and a request data item identifier. The requested data can be shared via RSU broadcast or other neighboring vehicles containing the data. The types of data items transmitted by vehicles are divided into emergency data items and other data items. The emergency data is very sensitive to time and needs to be transmitted to the target vehicle within the prescribed deadline.

In a network environment, each vehicle can communicate with its neighboring vehicles through Dedicated Short-Range Communication (DSRC) [25]. The network environment refers to IEEE802.11p. Vehicle communication uses multiple channels for communication, including a control channel and six service channels. The control channel is used to send management control information. Vehicles can send their basic information to neighboring vehicles and RSUs through the control channel. The service channel is used to send data transmission information and vehicles use the service channel for data transmission.

In Figure 1, all vehicles are covered by the RSU and each vehicle contains a cached data item and a request data item identifier. The underlined letters indicate the data items that the vehicle has cached and the underlined letters indicate the data identifier that the vehicle needs to request. For example, a $V_{1}$ vehicle needs to request data items $d_{1}$ and $d_{2}$, and a $V_{2}$ vehicle has cached data items $d_{2}$, $d_{3}$, and $d_{5}$. For example, there are two ways for $V_{4}$ to receive the data item $d_{2}$. The first way is to transmit the data item $d_{2}$ to $V_{4}$ through the V2I communication mode. In a second way, $V_{2}$ has buffered data item $d_{2}$, and $V_{2}$ can send data items $d_{2}$ to $V_{4}$ through the V2V communication mode. The data transmission collaborative scheduling algorithm maximizes the weighted service capacity in the scheduling period.

### 3.2. Multi-Channel Data Transmission Cooperative Scheduling Problem

Vehicle data items are divided into safety data items ED and other data items OD. ED is more sensitive to time than OD. Set the urgency of receiving data items of the vehicle $v_{i}$ to receive data items so that ED is transmitted to the required vehicles within a limited time. $\zeta_{v_{i}}$ is proportional to the number of ED in $v_{i}$. The sum of vehicle service weights ω through cooperative transmission in the scheduling period is shown in Equation (1), which means that to maximize the number of priority transmission of urgent data and the number of successfully received data items within the scheduling period.

(1) $$\omega \left(t\right)=\sum_{v_{i}\in \;\Omega_{I}^{\Upsilon }\left(t\right)⋃\Omega_{V}^{\Upsilon }\left(t\right)}\zeta_{v_{i}}\left(t\right)$$

In Equation (1), $\Omega_{I}^{\Upsilon }$ and $\Omega_{V}^{\Upsilon }$ respectively represent the set of vehicles receiving data items in V2I communication and the set of vehicles receiving data items in V2V. If $\left(\Omega_{I}^{\Upsilon },\Omega_{V}^{\Upsilon },\vartheta \right)$ is used as a scheduling scheme. The optimization goal of this paper is to maximize the weighted service capacity of all vehicles in the coverage area. This means finding the optimal transmission scheduling strategy that maximizes the weighted service capacity. The optimization model of the optimal transmission scheduling strategy is shown in Equation (2). Table 1 shows a summary of commonly used abbreviations and symbols in this paper.

(2) $$\left(\Omega_{I}^{\Upsilon },\Omega_{V}^{\Upsilon },\vartheta \right)*=\arg\max_{\left(\Omega_{I}^{\Upsilon },\Omega_{V}^{\Upsilon },\vartheta \right)\in \left(\Omega_{I}^{\Upsilon },\Omega_{V}^{\Upsilon },\vartheta ,\zeta \right)}\omega \left(t\right)$$

## 4. Multi-Channel Data Transmission Cooperative Scheduling Process

The interference graph is constructed according to the traffic scenario. The vertices in the graph represent a feasible transmission scheme of the vehicle receiving data items and the edges represent interference between transmission schemes. Then calculate the signal-to-noise ratios of the vertices in the interference graph and allocate channels to eliminate some interference. Obtaining the optimal schedule is equivalent to solving the Maximum Weighted Independent Set (MWIS) in the interference graph. MWIS is an NP-hard problem, therefore the multi-channel data transmission cooperative scheduling scheme is an NP-hard problem. In this paper, a solution based on the ISing model is used to solve the problem.

### 4.1. Constructing Interference Graph

For each vehicle $v_{i}\in V$, receiving data items of $v_{i}$ in V2I communication mode can be expressed as $\phi_{I}^{v_{i}}$ , $\phi_{I}^{v_{i}}\in R_{v_{i}}$. Then the receiving vehicle set $\Omega_{I}^{\Upsilon }$ in the V2I communication mode is expressed as:

(3) $$\Omega_{I}^{\Upsilon }=\left\{v_{i}|v_{i}\in V_{r}\wedge \phi \left(\Upsilon_{I}^{v_{i}}\right)\in R_{v_{i}}\right\}.$$

In the same period, both the sending vehicles ($v_{i}$) and receiving vehicles ($v_{j}$) are in V2V communication mode. The $v_{i}$ transmits $\phi_{V}^{v_{i}}$ to $v_{j}$. The $\phi_{V}^{v_{i}}$ must be data items that have been cached by the $v_{i}$ and that have been requested by the $v_{j}$, expressed as $\phi_{V}^{v_{i}}\in C_{v_{i}}\wedge \phi_{V}^{v_{i}}\in R_{v_{j}}$. The $v_{i}$ and $v_{j}$ need to be less than the vehicle’s one-hop communication distance, expressed as $v_{j}\in \eta_{v_{i}}$. The $v_{i}$ use the same channel for communication as $v_{j}$, expressed as $\vartheta_{v_{i}}=\vartheta_{v_{j}}$. The receiving vehicle set $\Omega_{V}^{\Upsilon }\left(\phi_{V}^{v_{i}}\right)$ of the data item $\phi_{V}^{v_{i}}$ is expressed as:

(4) $$\Omega_{V}^{\Upsilon }\left(\phi_{V}^{v_{i}}\right)=\left\{v_{j}|\phi_{V}^{v_{i}}\in C_{v_{i}}\wedge \phi_{V}^{v_{i}}\in R_{v_{j}}\wedge v_{j}\in \eta_{v_{i}}\wedge \vartheta_{v_{i}}=\vartheta_{v_{j}}\right\}.$$

The set of receiving vehicles in the V2V communication mode during the scheduling period *t* is $\Omega_{V}^{\Upsilon }\left(t\right)$, the expression is as follows:

(5) $$\Omega_{V}^{\Upsilon }\left(t\right)=\underset{\phi_{V}^{v_{i}}\in R_{v_{i}},v_{i}\in V}{⋃}\Omega_{V}^{\Upsilon }\left(\phi_{V}^{v_{i}}\right).$$

In V2V communication mode, the vehicles $v_{i}$,$v_{j}$ and $v_{k}$ are all in V2V mode. While sending vehicles $v_{i}$ and $v_{j}$ using the same channel, $v_{k}$ is within the one-hop communication distance of them, then $v_{k}$ receives interference from $v_{j}$ when receiving data. The interference set Γ for vehicles using the same channel to transmit data in the V2V communication mode is expressed as:

(6) $$\Gamma =\left\{v_{k}|v_{k}\in \eta_{v_{i}}\wedge v_{k}\in \eta_{v_{i}}\wedge \vartheta_{v_{i}}=\vartheta_{v_{j}}=\vartheta_{v_{k}}\wedge SINR\left(v_{i},v_{k}\right)<\beta \right\}.$$

**Figure 2:**
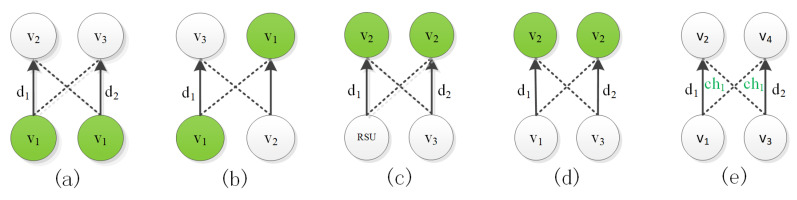
Interference example diagram. (**a**) The vehicle sending interference; (**b**) Vehicle sending and receiving interference; (**c**) Vehicle receiving communication mode interference; (**d**) The vehicle receiving interference; (**e**) Vehicle V2V communication interference.

Due to the following data transmission restrictions, different transmission schemes may interfere with each other. The solid line in Figure 2 represents the communication between two vehicles and the dotted line represents the conflict caused by the communication between the vehicles. d1 represents the data item sent by the node, and ch1 represents the channel used by the vehicle for communication.

**Constraint 1:** Two links $v_{s}\overset{d_{x}}{⟶}v_{r}$ and $v_{s}\overset{d_{x}}{⟶}v_{r^{\prime }}$. If the data items $d_{x}$ and $d_{y}$ are different, it means that the vehicle $v_{s}$ sends different data items in the same scheduling cycle. As shown in Figure 2a, there is interference that does not satisfy constraint 1 between $v_{1}\overset{d_{1}}{⟶}v_{2}$ and $v_{1}\overset{d_{2}}{⟶}v_{3}$, it violates the nature of the vehicle that can only send one type of data at the same time in the network environment.

**Constraint 2:** Two links $v_{s}\overset{d_{x}}{⟶}v_{r}$ and $v_{s^{\prime }}\overset{d_{y}}{⟶}v_{r^{\prime }}$. If vehicles $v_{s}$ and $v_{r^{\prime }}$ are the same or $v_{s^{\prime }}$ and $v_{r}$ are the same, it means that $v_{s}$ is both a sending vehicle and a receiving vehicle in the same scheduling period. As shown in Figure 2b, there is interference that does not satisfy constraint 2 between $v_{1}\overset{d_{1}}{⟶}v_{3}$ and $v_{2}\overset{d_{2}}{⟶}v_{1}$, it violates the nature of half-duplex communication of vehicles in the network environment.

**Constraint 3:** Two links ${\mathrm{RSU}}_{s}\overset{d_{x}}{⟶}v_{r}$ and $v_{s}\overset{d_{y}}{⟶}v_{r^{\prime }}$. If the vehicles $v_{r}$ and $v_{r^{\prime }}$ are the same, it means that they are in V2I and V2V communication modes in the same scheduling period. As shown in Figure 2c, there is interference that does not satisfy constraint 3 between the $\mathrm{RSU}\overset{d_{1}}{⟶}v_{2}$ and the $v_{3}\overset{d_{2}}{⟶}v_{2}$, it violates the nature of the vehicle in the network environment that the vehicle can only be in one communication mode at the same time.

**Constraint 4:** Two links $v_{s}\overset{d_{x}}{⟶}v_{r}$ and $v_{s^{\prime }}\overset{d_{y}}{⟶}v_{r}$ . If the data items $d_{x}$ and $d_{y}$ are different, it means that the $v_{r}$ receives two different data items in the same scheduling period. As shown in Figure 2d, there is interference that does not satisfy constraint 4 between $v_{1}\overset{d_{1}}{⟶}v_{2}$ and $v_{3}\overset{d_{2}}{⟶}v_{2}$, it violates the nature of vehicles that can only receive one type of data at the same time in the network environment.

**Constraint 5:** Two link $v_{s}\overset{d_{x}}{⟶}v_{r}$ and $v_{s^{\prime }}\overset{d_{y}}{⟶}v_{r^{\prime }}$, if $\vartheta_{v_{s}}=\vartheta_{v_{s^{\prime }}}=\vartheta_{v_{r}}=\vartheta_{v_{r^{\prime }}}$ and $v_{r}\in \eta_{v_{s^{\prime }}}$ or $v_{r^{\prime }}\in \eta_{v_{s}}$, it means that $v_{r}$ will be interference by $v_{s^{\prime }}$ when receiving data items sent by $v_{s}$. As shown in Figure 2e, there is interference that does not satisfy constraint 5 between $v_{1}\overset{ch_{1}}{\underset{d_{1}}{\to }}v_{2}$ and $v_{3}\overset{ch_{1}}{\underset{d_{2}}{\to }}v_{4}$. That is, sending a message from a sending vehicle will interfere with the receiving vehicle.

Construct the interference graph $G_{I}$ through the following steps: (1) Constructing vertexes of the transmission scheme in the interference graph in the V2I communication mode through the above set $\Omega_{I}^{\Upsilon }$ and $\phi_{I}^{v_{i}}$; (2) construct vertices of the transmission scheme in the V2V communication mode by using the set $\Omega_{V}^{\Upsilon }$ and $\phi_{V}^{v_{i}}$ vertex; (3) if any two vertexes do not meet the above five constraints, add an edge between the two vertices; and (4) calculate the weight of each vertex. The weight represents the urgency to receive the data items of the receiving vehicle, as shown in Equation (7). After the above 4 steps, an interference pattern $G_{I}=\left(U_{I},E_{I}\right)$ is formed.

(7) $$\xi_{v_{i}}={\left\{\frac{\left(\lambda \times |ED_{v_{i}}|+|OD_{v_{i}}|\right)}{dis_{v_{i}}}\times vel_{v_{i}}\right\}}^{\chi }$$

### 4.2. Channel Assignment Algorithm

According to the actual traffic scenario, we construct an initial interference graph $G_{I}=\left(U_{I},E_{I}\right)$. Before to channel allocation, the RSU calculates the signal-to-noise ratio SINR of each vertex. This is shown in Equation (8).

(8) $$SINR\left(v_{s},v_{r}\right)=\frac{P_{v_{s}}\times dist{\left(v_{s},v_{r}\right)}^{-\alpha }}{\sum_{v_{s^{\prime }}\in V\left(t\right)\wedge s^{\prime }\neq s}P_{v_{s^{\prime }}}\times dist{\left(v_{s^{\prime }},v_{r}\right)}^{-\alpha }+N_{0}}$$

$P_{v_{s}}$ are the transmission power of the vehicle $v_{s}$; $dist(v_{s},v_{r})$ is the distance between the sending and the receiving vehicle; α>0 is the path loss parameter; and $N_{0}\geq 0$ represents the background noise value. While the target vehicle SINR is greater than the set threshold β, it can be considered that the target vehicle successfully receive data items. We use the channel allocation algorithm to assign channels to the vertices of ${G_{I}}^{\prime }$. $ch_{i}$ indicates available channels and the satisfied constraints are as shown in Equation (9). The process of the channel allocation algorithm is shown in Algorithm 1.

(9) $$\left\{\begin{array}{c} \vartheta_{v_{i}}=ch_{i},i=1,2,\ldots ,7\\ 7\geq ch_{i}\geq 1\\ \vartheta_{v_{i}}\neq \vartheta_{v_{i}},if\left(v_{i},v_{j}\right)\in E_{S} \end{array}\right.$$

**Algorithm 1:** Channel Allocation.

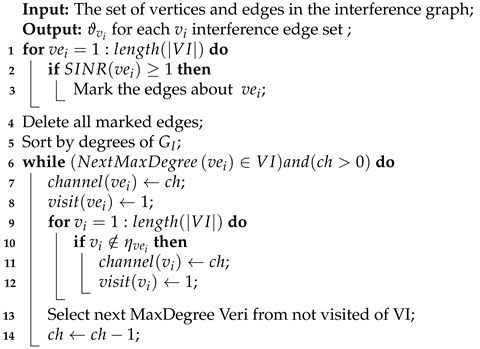



Algorithm 1 allocates channels based on SINR. First, by calculating the signal-to-noise ratio of each vertex, the interference edges that meet the communication conditions are deleted (Algorithm 1, 1–6 lines). Then select the vertex with the largest degree in $G_{I}$, assign the same channel to its non-adjacent vertexes, delete the corresponding interference edges, and reduce the number of channels by 1. Finally, select the vertexes that have not been accessed and have the highest degree, and repeat the above steps until all the vertexes are accessed or the number of channels is 0 (Algorithm 1, 7–20 lines). If the number of channels is insufficient, the conflicting edges in $G_{I}$ can be retained and the interference problems can be solved by scheduling in Algorithm 2.

### 4.3. Generate Schedule Scheme

The data transmission collaborative scheduling scheme is equivalent to the MWIS in $G_{I}$. It is to find the most optimal independent set $Z^{*}$, which needs to be satisfied $\forall Z\subseteq U_{s}$ and $\zeta \left(Z^{*}\right)\geq \zeta \left(Z\right)$, as shown in Formula 10. Formula 10 is the expression formula for optimizing the weighted service capacity in the target scheduling period in this paper.

(10) $$\begin{array}{c} \max\sum_{v_{i}\in Z}\zeta_{v_{i}}\\ s.t.\left\{\begin{array}{c} Z\subseteq U_{s}\\ \left(v_{i},v_{j}\right)\notin E_{s},\forall v_{i},\forall v_{j}\in Z,v_{i}\neq v_{j} \end{array}\right. \end{array}$$

The solution to the maximum weighted independent set is based on the ISing model. The idea comes from the law of the motion of the molecules in the metal. It divides the transmission cycle into several time slots, and each time slot has an H(δ) corresponding comparison with the value generated by the previous time slot. If the current value is greater than the value of the previous time slot, change the corresponding parameter, otherwise do not change. After multiple iterations, the minimum H(δ) is finally obtained, as shown in Equation (11):

(11) $$H\left(\delta_{i}\right)=A\sum_{(i,j)\in E,i<j}x_{i}x_{j}-\sum_{i=1}^{N}\zeta_{v_{i}}\zeta_{v_{j}}$$

(12) $$x_{i}=\frac{\delta_{i}+2}{2},\;\delta_{i}=\left\{\pm 1\right\}$$

The maximum weighted independent set is obtained by the algorithm. The vertexes in the independent set indicate that the scheduling operation does not interfere with each other in the same cycle. The vertexes not in the set will be scheduled in the next cycle. Then in the next cycle, the service queue is updated according to the joining and leaving of the vehicle. The data transmission scheduling algorithm is specifically shown in Algorithm 2.
**Algorithm 2:** ISing-Based Greedy Selection.
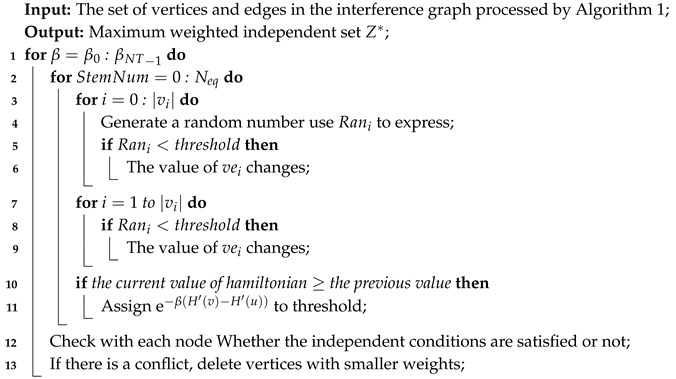


Algorithm 2 initially generates a random value for each vertex (between 0–1). If this random number is less than the set threshold, the current vertex value changes, otherwise it is unchanged (Algorithm 2, 3–7 lines). Then judge whether all the adjacent vertices of the current vertex are all 0 or all random numbers are less than the current random number. If the above conditions are met, the current value state changes (Algorithm 2, 8–13 lines). Secondly, it is judged whether the current value H(δ) is smaller than the previous one at the end of each iteration. If it is smaller, all vertexes states are maintained, otherwise the threshold is re-assigned. Finally, execute the program Nep loops at each tiny time slice to achieve the system steady-state (Algorithm 2, 14–20 lines).

### 4.4. Scheduling Process Analysis

Assume that the total number of vehicles in the network is |V|, the total number of data items requested by the vehicles is |Q|, and the total number of conflicting edges is |EC|. The complexity of Algorithm 1 to construct the initial interference graph is $O(|V{|}^{2})$, and the complexity of calculating the SINR of all vertexes is O(|V|). In the worst case of the channel allocation phase, each vertex in the graph needs to determine whether another vertex is its adjacent vertex. The complexity is $O(|V{|}^{2}+|V|)$, therefore the complexity of Algorithm 1 is $O(|V{|}^{2})$. In Algorithm 2, the complexity of sending a message by each vertex is O(|V|×|EC|), and each vertex judges the state of its neighboring vertices as well. The total overhead of the Algorithm 2 is $O(C^{2}\times \left(|V\right|\times \left|EC|)\right)$ (*C* is the number of time slots divided and the constant number of iterations in the Algorithm 2), and the time complexity of Algorithm 2 is O((|V|×|EC|)). That is, the complexity of the MDTCS algorithm is O((|V|×|EC|)).

This paper studies the V2I / V2V cooperative communication to solve the data transmission scheduling problem with the support of RSU. The optimization goal is to maximize the weighted service capacity of all vehicles in the coverage area. A three-stage data transmission cooperative scheduling mechanism was proposed to realize the cooperative communication of vehicles through V2I/V2V with the assistance of RSU. (1) All vehicle though control channels in the RSU coverage area send Basic Safety Messages (BSM), and each vehicle can obtain the BSM of its neighboring vehicle. (2) The vehicle sends an environmental awareness message to the RSU through the control channel. RSU can get the information of all vehicles in the area. (3) The RSU obtains the optimal data transmission scheme of V2I / V2V cooperative communication based on the obtained information. In the end, each vehicle chooses V2I or V2V communication mode to transmit corresponding data according to the scheduling decision, and the vehicle transmission data does not interfere with each other in the same scheduling cycle.

Vehicles that have failed to successfully transmit data in the current dispatch period. As each vehicle has a degree of urgency, the degree of urgency is directly proportional to the weight coefficient and inversely proportional to the remaining driving time of the vehicle in the RSU. This ensures that vehicles that have not successfully received data items in the current cycle have a higher urgency in the next cycle. As time increases, the priority becomes higher, thereby ensuring that these vehicles with a long waiting time can successfully transmit data.

The above three phases are all completed in one scheduling cycle. DSRC attaches the requested data items to the BSM, and the capacity of the BSM and the requested information contains about 54 B [23]. In a typical intelligent transportation system, the size of the data item is 500 kb, and the minimum transmission rate is 6 Mb/s, so the maximum transmission time is about 0.72 s. In this paper, the transmission scheduling period is set to 1 s, so the three phases can be completed in one cycle.

## 5. Performance Evaluation

### 5.1. Experimental Environment

This section evaluates the overall performance of the MDTCS algorithm from multiple perspectives. First, the detailed information of the VANETs scene is listed, that the traffic data is generated by Simulating Urban Mobility (SUMO) to ensure the effectiveness of the simulation [26]. Then, this section lists a series of performance indicators to explain the stability of the MDTCS algorithm on data transmission communication. A series of performance indicators will be explained in detail in the following sections. Finally, the MDTCS algorithm proposed in this paper is compared with the IF algorithm in [27], the CDD algorithm in [23], and the MCV-DTS algorithm [24] in simulation experiments. The application scenarios of this article and the other three algorithms are very similar. The IF algorithm is a classic algorithm for V2I single-channel communication under the coverage of RSU. Vehicles in its coverage area can only receive data through RSU broadcasts. The other three algorithms are algorithms that use V2I/V2V cooperative communication.

The simulated road scenario uses a two-way, six-lane straight road with the same parameters on the opposite lanes. As the density of traffic increases, the average speed of vehicles on the road decreases. According to the DSRC standard, the effective transmission radius of the RSU is 500 m and the effective transmission radius of the vehicle unit (on-board unit) OBU is 150 m. As shown in Table 2, six different traffic environments are simulated.

This paper quantitatively analyzes the performance of the algorithm according to the following four indicators.

- **Weighted Service Capacity:** The sum of the weights of the target vehicles that have successfully received the data items in the complete scheduling cycle;
- **Average service proportion:** In the complete cycle, the ratio of data items that a vehicle successfully transmits using V2I communication to that using V2V communication;
- **Completion Probability:** The ratio of the number of data items successfully received by the vehicle to the total number of data items requested by the vehicle;
- **Service delay:** The time from a request to submit a data item to complete a data service of a vehicle during a complete dispatch cycle.

### 5.2. Simulation Results

**Figure 3:**
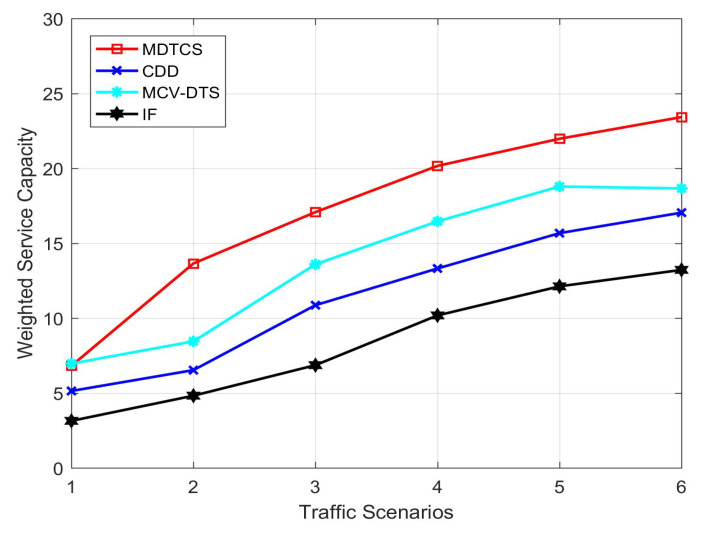
Weighted service capacity of the four algorithms.

Figure 3 shows the weighted service capacity of four algorithms increases when the vehicle density increases. When the traffic density is low, there is not much difference between the weighted service capacity value of the algorithm in this paper and the MCV-DTS algorithm. Since the other two algorithms use single-channel communication and the IF algorithm uses the V2I communication mode, the IF algorithm has a lower weighted service capacity value than the CDD algorithm. It can be seen from the simulation results that the MDTCS algorithm, MCV-DTS algorithm, and CDD algorithm in this article are significantly better than the IF algorithm in different scenarios. As the vehicle received data in the IF algorithm can only be broadcast through RSU, which greatly limits the transmission of vehicles. The CDD algorithm does not consider multi-channel communication and the algorithm does not show good performance. Based on the multi-channel data transmission coordinated scheduling mechanism, the MDTCS algorithm completes data transmission according to the urgency priority policy of the data item, which improves the system’s weighted service capacity.

**Figure 4:**
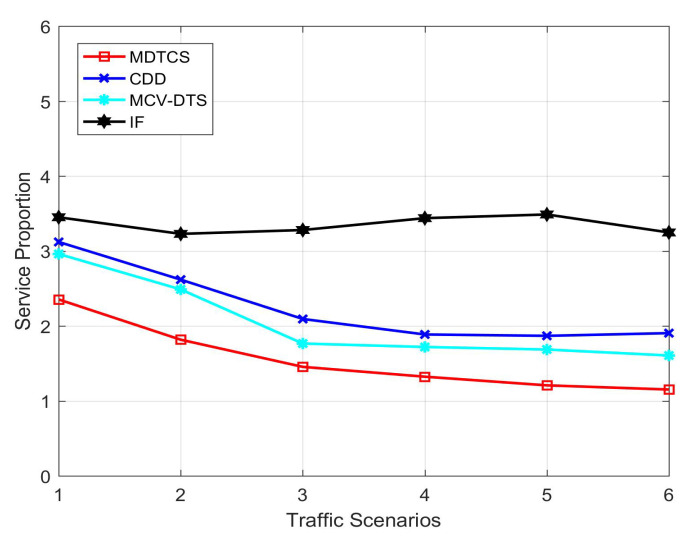
Average service proportion of the four algorithms.

Figure 4 shows the service proportion of the proposed MDTCS algorithm and the other three algorithms in different traffic scenarios. When the traffic volume is small, the system needs to transmit fewer data items. All four algorithms can use RSU to broadcast a lot of data items to vehicles in the coverage area, so the ratio of RSU broadcast data items to V2V communication services is high. As the traffic volume increases, the three algorithms restrict V2I communication through the cooperative scheduling mechanism, so the service ratio decreases. As shown in the figure, the service ratio of the IF algorithm is very high, because the IF algorithm can only meet a small part of data requests, which shows that the system service ratio is high. When the network scale complexity reaches a certain level, the number of vertices in the graph increases exponentially, and the algorithm for collaborative scheduling processing shows limitations, and the average service ratio of the algorithm approaches a fixed value.

**Figure 5:**
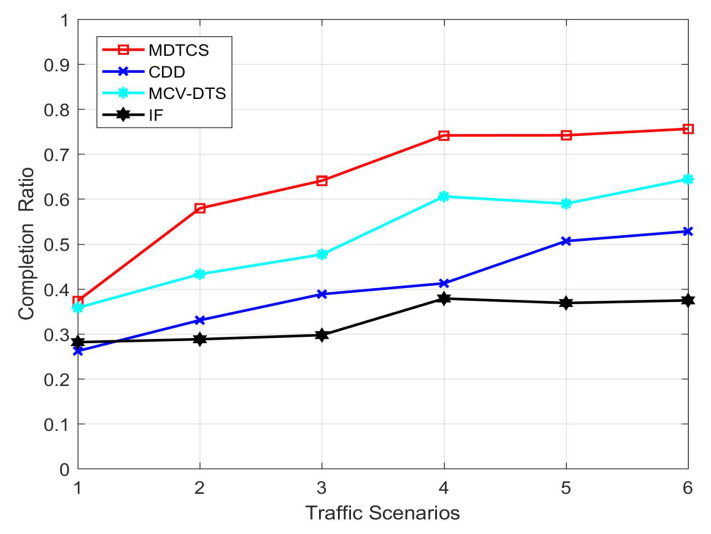
Completion ratio of the four algorithms.

Figure 5 shows the system request completion for the proposed MDTCS algorithm and other three algorithms in different traffic scenarios. It can be seen from the figure that the system request completion degree increases with the increase of vehicle density. As the vehicle density is small and the speed of the vehicle is high, the time spent in the service area is short, so the system request completion is low. When the vehicle density increases, the vehicles stay in the service area for a longer time, which increases the scheduling cycle. Eventually the system request completion increases. In the figure, the MDTCS algorithm has a better request completion than the other three algorithms.

**Figure 6:**
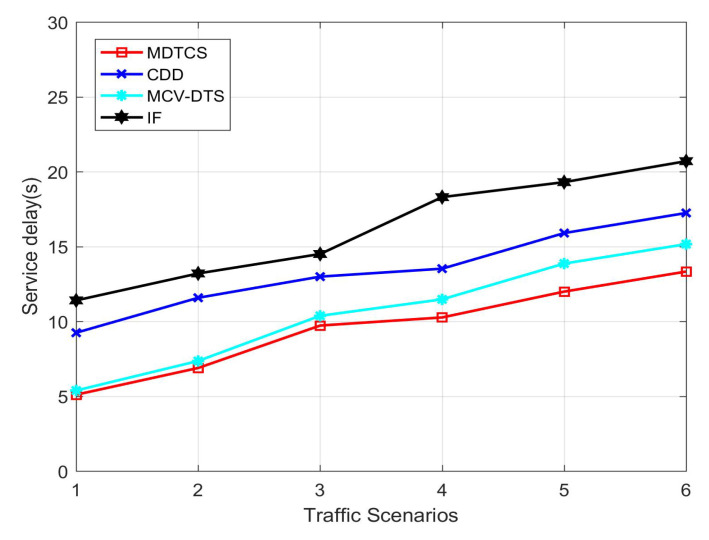
Service delay of the four algorithms.

Figure 6 shows the service delay of the proposed MDTCS algorithm and the other three algorithms in different traffic scenarios. The initial vehicle density is small and the delays shown by several algorithms are relatively small and close. This is due to the large proportion of V2I communication and the performance of this algorithm is not well reflected. When the vehicle density increases, the MDTCS algorithm, CDD algorithm, and MDTCS algorithm proposed in this paper make V2I communication and V2V communication transmit concurrently through a coordinated data scheduling transmission mechanism. Based on this, the MDTCS algorithm adds channel allocation based on the signal-to-noise ratio, which greatly alleviates the situation of insufficient channels, and the data scheduling and allocation algorithm based on the ISing model reduces the waiting time of vehicle services, showing a lower latency than other algorithms.

As the above simulation results show, the MDCTS algorithm proposed in this paper is better than the other three algorithms in terms of weighted service capacity, average service proportion, completion probability, and service proportion performance. In a dense traffic scene, the network service capacity has increased significantly and service delays have effectively decreased, which ensures that emergency data is preferentially transmitted to the target vehicle and the maximum weighted service capacity of the network.

## 6. Conclusions

Aiming at the problem of cooperative scheduling of V2I and V2V data transmission supported by RSU, this paper proposes an MDTCS data transmission scheduling algorithm based on the signal-to-noise ratio. The algorithm constructed an interference graph of the data transmission scheme according to the five constraints. Due to the lack of channel resources, the SINR-based channel allocation algorithm was used to assign different channels to the vertices of conflicting edges, which greatly reduced the waste of channel resources. Then, a greedy algorithm based on the ISing model was used to mark the urgency of the requested data items, and the data items with high urgency were sent preferentially. Finally, the optimal transmission strategy was obtained through the greedy algorithm. Simulation results showed that compared with the other three algorithms, when the traffic volume was intensive, the service capacity and service delay were significantly improved. Our goal in the next stage is to improve the overall service capacity and effectively reduce the service delay and other performance in the non-intensive area of traffic flow.

## Figures and Tables

**Table 1:** Summary of notations.

Notations	Descriptions
*V*	set of vehicles $V=\left\{v_{1},v_{2},\ldots ,v_{n}\right\}$
$\phi_{I}^{v_{i}}$	$v_{i}$ receive a set of data items in V2I
$\Omega_{I}^{\Upsilon }$	receive a set of vehicles in V2I
$\Omega_{V}^{\Upsilon }$	receive a set of vehicles in V2V
ED	emergency data item
OD	other data item
$C_{v_{i}}$	a set of cached data items of $v_{i}$
$R_{v_{i}}$	a set of request data items of $v_{i}$
$\vartheta_{v_{i}}$	channel number used by $v_{i}$
$\eta_{v_{i}}$	a set of neighboring vehicles set of $v_{i}$
$SINR(v_{i})$	the SINR value of $v_{i}$
$\zeta_{v_{i}}$	urgency of $v_{i}$
$G_{I}$	interference graph $G_{I}=\left(U_{c},E_{c}\right)$
$Z^{*}$	maximum weighted independent set
$dis_{v_{i}}$	the distance from the $v_{i}$ to the RSU boundary
$vel_{v_{i}}$	driving speed of $v_{i}$

**Table 2:** Traffic simulation parameters.

Traffic Scenarios	Mean Velocity (km/h)			Mean Density (Vehicles/km)		
	Line 1	Line 2	Line 3	Line 1	Line 2	Line 3
1	103.8	82.36	71.58	12.96	12.47	11.85
2	99.54	80.13	64.32	17.36	18.56	18.22
3	92.12	74.55	54.23	22.11	26.16	30.56
4	81.45	73.25	40.56	30.55	39.48	40.72
5	65.49	38.76	30.16	41.45	57.13	65.39
6	53.12	29.14	21.56	49.82	67.55	73.94
